# Prevalence of Major Cardiovascular Disease Events Among People Diagnosed With Schizophrenia Who Have Sleep Disturbance, Sedentary Behavior, or Muscular Weakness

**DOI:** 10.1093/schizbullopen/sgaa069

**Published:** 2021-01-19

**Authors:** Alexandra Berry, Alison R Yung, Matthew J Carr, Roger T Webb, Darren M Ashcroft, Joseph Firth, Richard J Drake

**Affiliations:** 1 Division of Psychology & Mental Health, School of Health Sciences, Faculty of Biology, Medicine and Health, University of Manchester, Manchester Academic Health Sciences Centre (MAHSC), Manchester, UK; 2 Centre for Youth Mental Health, The University of Melbourne, Parkville, Victoria, Australia; 3 Division of Pharmacy & Optometry, School of Health Sciences, Faculty of Biology, Medicine and Health, University of Manchester, Manchester Academic Health Sciences Centre (MAHSC), Manchester, UK; 4 NIHR Greater Manchester Patient Safety Translational Research Centre, University of Manchester, Manchester, UK; 5 Greater Manchester Mental Health NHS Foundation Trust, Manchester, UK

**Keywords:** severe mental illness, grip strength, morbidity, psychotic disorder, physical illness, lifestyle behaviors

## Abstract

**Objective:**

To estimate prevalence of major cardiovascular events among people with schizophrenia who had experience of sleep disturbance, sedentary behavior or muscular weakness, and assess evidence for raised prevalence in these individuals compared to people with schizophrenia without these characteristics.

**Methods:**

UK Biobank data on individuals diagnosed with schizophrenia (*n* = 1544) were used to examine the prevalence of major cardiovascular events, specifically myocardial infarction, stroke, heart failure and cardiovascular death, among participants with candidate risk factors. Generalized linear models were fitted to estimate prevalence ratios (PRs) for major cardiovascular events among participants with self-reported sleep disturbance, self-reported sedentary behavior, and muscular weakness measured using a handgrip dynamometer. These ratios were adjusted for QRISK3 score—a validated cardiovascular risk prediction algorithm for the UK population.

**Results:**

Prevalence of major cardiovascular events was significantly higher among participants with daytime sleepiness, independent of QRISK3 score, and snoring, a proxy for sleep-disordered breathing (adjusted PR 1.26; 95% CI 1.03, 1.55, *P* = .03). Prevalence was also independently higher among participants with low muscular strength (adjusted PR1.36; 95% CI 1.05, 1.75, *P* = .02). The adjusted prevalence ratios among participants with short or prolonged sleep duration, insomnia, or sedentary behavior did not indicate independently raised prevalence among these groups.

**Conclusion:**

Prevalence of major cardiovascular events among people with schizophrenia was higher in participants with muscular weakness and sleep disturbance evidenced by daytime sleepiness. Further research is required to determine how these factors can be routinely identified and addressed in the clinical management of cardiovascular risk among patients with schizophrenia.

## Introduction

People diagnosed with a severe mental illness (SMI), such as schizophrenia, have a shorter lifespan than the general population by 10 to 20 years.^1^ The most common cause of death in this group is cardiovascular disease (CVD).^2^ Compared with the general population, people with schizophrenia have a higher incidence of CVD and an elevated mortality risk due to CVD.^2,3^ They often have multiple risk factors for CVD, including potential risks from antipsychotic medication usage,^4^ obesity,^5^ type 2 diabetes,^6^ and dyslipidemia and hypertension.^7^ As increased risk of cardiometabolic diseases in SMI populations can arise even prior to antipsychotic treatment,^8^ lifestyle factors such as elevated rates of smoking^9^ and poor diet^10^ are also likely to play a salient role.

To estimate an individual’s absolute risk of developing CVD, risk calculator algorithms have been devised for application in routine clinical practice.^11^ Previously the QRISK2 calculator was recommended for use in the United Kingdom,^12,13^ which included body mass index (BMI), diabetes, hypertension, lipid measurements, and smoking status within the algorithm.^14^ However, it did not consider SMI diagnosis or second-generation antipsychotic use^14^ and therefore likely underestimated CVD risk in SMI populations.^12^ The updated QRISK3 calculator is now the tool of preference for clinical use in the United Kingdom^15^ and includes SMI diagnosis and second-generation antipsychotic medication usage.^16^

Despite these improvements, evidence is accumulating for other potential risk factors for CVD that are not captured in these risk algorithms, but that are disproportionately associated with SMIs. For example, sleep disturbances are not considered in current CVD risk assessments, even though these are known to contribute to the etiology of myocardial infarction (MI).^17,18^ Sleep disturbances are of raised prevalence in SMI.^19–21^ Furthermore, “sedentary behavior,” which is defined as time spent in a sitting, reclining, or lying position during waking hours with energy expenditure less than or equal to 1.5 metabolic equivalents of task (METs),^22^ is also heightened among persons with SMI.^23^ Such behavior has been shown to independently increase the risk for CVD onset and subsequent related mortality in the general population.^24^ Similarly, grip strength (measured as a proxy for muscular strength) provides a strong indicator of cardiovascular risk in the general population,^25^ and is much lower among individuals with schizophrenia.^26,27^ However, again, these factors are overlooked in current risk assessments for CVD in SMI.

We therefore aimed to estimate the prevalence of major cardiovascular events among people diagnosed with schizophrenia who had sleep disturbance, sedentary behavior, or muscular weakness. We hypothesized that major cardiovascular events would be more prevalent among participants with sleep disturbance, sedentary behavior, and muscular weakness, independent of QRISK3 score.

## Methods

### UK Biobank

This is a large population-based cohort study with *N* = 503 325 participants who were recruited across the United Kingdom from 22 assessment centers between 2006 and 2010. Ethical approvals were gained from the North West Multi-Centre Research Ethnics Committee and the National Health Service (NHS) National Research Ethics Service. The detailed protocol, scientific rationale, and study design are published online.^28^ Assessments completed at baseline included signed consent, computer-assisted interview, self-completed touch-screen questionnaire, physical and functional measures, and blood, urine, and saliva sample collection.^28,29^

### Study Design

Participants sampled from the UK Biobank for this study had all been diagnosed with schizophrenia or a related psychotic disorder as classified by the International Classification of Diseases (ICD)-10 F20-F29 diagnoses.^30^ In a cross-sectional design we compared prevalence of major cardiovascular events among people with schizophrenia who also had each of the examined candidate risk factors (sleep disturbance, sedentary behavior, or muscular weakness) against people with schizophrenia without the candidate risk factor being considered.

### Assessment of Major Cardiovascular Events

We defined major cardiovascular events according to the ICD-10: that is a primary or secondary diagnosis of congestive heart failure or the following major adverse cardiovascular events (MACEs): fatal and non-fatal MI, fatal and non-fatal stroke, and cardiovascular death.^31^

### Assessment of Candidate Risk Factors

#### Sleep Disturbance.

This was defined based on self-reported sleep duration, insomnia, and daytime sleepiness. For sleep duration, participants were asked, “About how many hours sleep do you get in every 24 hours? (please include naps),” with responses in hourly increments. Total sleep time was categorized into “long sleep” (9 hours or longer), “adequate sleep” (7–8 hours), “short sleep” (5–6 hours) or “very short sleep” (4 hours or shorter).^32^

To assess insomnia, participants were asked, “Do you have trouble falling asleep at night or do you wake up in the middle of the night?” with responses of “never/rarely,” “sometimes,” “usually” and “prefer not to answer.” We categorized insomnia as a binary variable: “no evidence of insomnia” and “evidence of insomnia.”

To assess daytime sleepiness, participants were asked, “How likely are you to doze off or fall asleep during the daytime when you don’t mean to? (eg, when working, reading or driving)” with responses of “never/rarely,” “sometimes,” “often,” “all the time,” “do not know” and “prefer not to answer.” We categorized daytime sleepiness as a binary variable: “no evidence of daytime sleepiness” and “evidence of daytime sleepiness.” Previous studies have shown daytime sleepiness is often a consequence of sleep-disordered breathing,^33–35^ and people with schizophrenia have a raised prevalence of this condition.^36,37^ Therefore, when examining daytime sleepiness, we adjusted for snoring; the most common symptom of sleep-disordered breathing.^38^ Participants were asked, “Does your partner or a close relative or friend complain about your snoring?” with responses of “yes,” “no,” or “don’t know”.

#### Sedentary Time.

This was defined based on self-reported data regarding time spent during waking hours with energy expenditure of 1.5 METs or lower. Assessment was based on adapted questions from the validated short International Physical Activity Questionnaire (IPAQ).^39^ Specifically, participants were asked, “In a typical day, how many hours do you spend watching television?” and “In a typical day, how many hours do you spend using the computer?” We combined these answers into one variable for total time spent sedentary and split this into tertiles to reflect “low,” “moderate,” and “high” screen time.

#### Muscular Strength.

Grip strength, as a proxy for muscular strength, was measured in kilograms using a Jamar J00105 hydraulic hand dynamometer. Isometric grip force was assessed from a single 3-s maximal grip effort of the right- and left-side arms with participants seated upright with their elbow by their side and flexed at 90° so that their forearm was facing forward and resting on an armrest. The mean of the right- and left-side values was used, a method previously reported elsewhere.^40,41^ To consider biological differences in grip strength within sex and age-groups, we derived age- and sex-specific tertiles to reflect “low,” “moderate,” and “high” muscular strength.

### Assessment of QRISK3 Score

CVD risk calculators are designed for use with people with no prior history of CVD to predict their future risk and are not developed to predict future risk of major cardiovascular events in those who have already experienced a past event.^16,42^ Here we generated QRISK3 scores^16^ for all participants, with and without prior CVD history, as a means of coalescing the multiple established risk factors for CVD into a single composite score, as opposed to predicting future risk. The candidate risk factors of interest in this study could then be assessed for evidence of their prevalence being independently raised, with adjustment for multiple factors using just one covariate. The QRISK3 algorithm takes into consideration age, family history of CVD, gender, ethnicity, Townsend deprivation index, as well as some physical health comorbidities, prescribed medications and unhealthy lifestyle behaviors known to be associated with raised CVD risk.^16^ Missing values for continuous variables were imputed using the sample’s mean. Missing values for binary variables were not imputed, so as not to assume higher or lower risk without evidence.

### Statistical Analysis

Data were analyzed using STATA (version 14; Statacorp).^43^ Sociodemographic and clinical characteristics of the study sample were reported as numerators, denominators, and percentages. We performed a cross-sectional analysis to estimate the prevalence of major cardiovascular events among people with schizophrenia who had sleep disturbance, sedentary behavior, or muscular weakness. Poisson regression models with a robust sandwich variance were fitted to estimate prevalence ratios (PRs), 95% confidence intervals (CIs), Akaike’s information criterion (AIC), and Bayesian information criterion (BIC), adjusted for a 10 centile increase in the QRISK3 score as a continuous measure. Statistical significance was set at *P* = .05 (2-sided).

## Results

### Characteristics of the Study Sample

The cross-sectional study included 1544 participants diagnosed with schizophrenia, of whom 292 individuals had experienced major cardiovascular events and 1252 had not. Baseline characteristics are shown in table 1. The median age for the whole sample was 55 years (IQR 15) and 54% were male. Around a quarter of participants had a normal BMI, with 35% being either obese (BMI 30 kg/m^2^ or greater) or severely obese (BMI 40 or greater). Prevalence of sleep disturbance was raised; over 3 quarters of the sample had evidence of very short, short, or long sleep. Over a third of participants also had self-reported daytime sleepiness and over 3 quarters had self-reported insomnia (table 2). Mean muscular strength was substantially lower for the overall sample compared to UK sex- and age-specific normative data.^44^

**Table 1.** Sociodemographic and Clinical Characteristics for the Whole Study Sample: *N* = 1544

	*N*	%
Gender		
Male	836	54.1
Female	708	45.9
Ethnicity		
White British	1322	85.6
Black African	33	2.1
Indian	20	1.3
Other ethnic groups	138	8.9
Employment		
Paid employment/self-employed	316	21.0
Unable to work due to sickness/disability	606	40.3
Retired	416	27.7
Looking after home/family	46	3.1
Unpaid/voluntary work	28	1.9
Unemployed	80	5.3
Student	12	0.8
Smoking		
Current smoker	446	28.9
Ex-smoker	440	28.5
Never smoked	637	41.3
BMI		
Underweight (<18.5)	21	1.4
Normal (18.5–24.99)	393	25.5
Overweight (25–29.99)	561	36.3
Obese (30–39.99)	472	30.6
Severe obesity (≥40)	62	4.0
Total (serum) cholesterol		
≤5 mmol/L	546	35.4
>5 mmol/L	851	55.1
Systolic blood pressure		
<120 mmHg	324	21.0
≥120–139 mmHg	604	30.1
≥140 mmHg	533	34.5
Type 2 diabetes		
Yes	294	19.0
No	1250	81.0

*Note:* BMI, body mass index.

**Table 2.** Prevalence (%) Values and Unadjusted Prevalence Ratios (PRs) for Factors Associated With Major Cardiovascular Events Among People Diagnosed With Schizophrenia

		Major Cardiovascular Events Prevalence			
	*n*	*N*	%	PR (95% CI)	*P*
Insomnia					
Yes	245	1224	20.0	1.37 (1.02–1.85)	.04
No	44	302	14.6	(ref.: 100)	
Sleep duration (h)					
Very short sleep (≤4)	19	73	26.0	1.51 (0.99–2.30)	.05
Short sleep (5–6)	77	338	22.8	1.32 (1.03–1.71)	.03
Adequate sleep (7–8)	122	709	17.2	(ref.: 100)	
Long sleep (≥9)	67	375	17.9	1.04 (0.79–1.36)	.79
Daytime sleepiness					
Yes	123	544	22.6	1.35 (1.09–1.66)	.01
No	158	940	16.8	(ref.: 100)	
Screen time (h)					
Low	99	511	19.4	(ref.: 100)	
Moderate	116	653	17.8	0.92 (0.72–1.17)	.48
High	72	349	20.6	1.06 (0.81–1.40)	.65
Muscular strength (kg)					
Low	123	546	22.5	1.44 (1.11–1.87)	.01
Moderate	91	513	17.7	1.13 (0.85–1.50)	.39
High	73	466	15.7	(ref.: 100)	

*Note:* CI, confidence interval; PR, prevalence ratio.

### Unadjusted Prevalence Ratios and With Univariate Prevalence Ratios Adjustment for QRISK3 Score

Unadjusted prevalence ratios for the candidate risk factors are shown in table 2. Prevalence of major cardiovascular events was raised among participants with sleep disturbance, including self-reported history of insomnia (PR 1.37; 95% CI 1.02, 1.85), self-reported history of daytime sleepiness (PR 1.35; 95% CI 1.09, 1.66), self-reported very short sleep duration (PR 1.51; 95% CI 0.99, 2.30) and self-reported short sleep duration (PR 1.32; 95% CI 1.03, 1.71). Prevalence of major cardiovascular events was also raised among participants with low muscular strength (PR 1.44; 95% CI 1.11, 1.87). There was no evidence of significantly raised prevalence among participants with sedentary behavior or long sleep duration.

The baseline model (QRISK3 score) and subsequent multivariable models examining the prevalence of major cardiovascular events among people with schizophrenia who had the candidate risk factors, with adjustment for QRISK3 score, are shown in table 3. Prevalence was significantly elevated among participants with daytime sleepiness, independent of QRISK3 score and snoring (PR 1.26; 95% CI 1.03, 1.55), and was also significantly raised among participants with low muscular strength, independent of QRISK3 score (PR 1.36; 95% CI 1.05, 1.75). With adjustment for QRISK3 score, there was no evidence of significantly raised prevalence among participants with insomnia, very short, short or long sleep duration, or sedentary behavior.

**Table 3.** Prevalence Ratios (PRs) With Confounder Adjustment Using the Composite QRISK3 Score

Baseline model	Crude PR (95% CI)	*P*	AIC	BIC
QRISK3	1.30 (1.25–1.35)	<.001	1483.82	1494.51
Models	Adjusted PR^a,b^ (95% CI)	*P*	AIC	BIC
Insomnia	1.28 (0.96–1.70)	.09	1466.53	1482.52
Sleep duration			1447.08	1473.63
Very short sleep	1.29 (0.85–1.95)	.24		
Short sleep	1.20 (0.94–1.53)	.15		
Long sleep	1.00 (0.77–1.30)	.99		
Daytime sleepiness^c^	1.26 (1.03–1.55)	.03	1425.06	1451.58
Screen time (h)			1457.68	1478.97
Moderate	0.92 (0.73–1.15)	.46		
High	0.98 (0.75–1.27)	.86		
Muscular strength (kg)			1457.87	1479.19
Low	1.36 (1.05–1.75)	.02		
Moderate	1.17 (0.89–1.54)	.25		

*Note:* AIC, Akaike’s information criterion; BIC, Bayesian information criterion; CI, confidence interval; PR, prevalence ratio.

^a^Prevalence ratio for 10 centile increase in the QRISK3 score.

^b^Adjusted for QRISK3 score.

^c^Adjusted for snoring.

## Discussion

We investigated whether prevalence of major cardiovascular events was raised among participants with schizophrenia who had sleep disturbance, sedentary behavior, and muscular weakness, independent of other known CVD risk factors. Our findings indicate that people with schizophrenia with self-reported daytime sleepiness have a raised prevalence of major cardiovascular events, independent of other known risk factors; ie, those within the QRISK3 algorithm^16^ and snoring, which we used as a marker for sleep-disordered breathing. Common causes of daytime sleepiness include irregular or insufficient sleep at night, resulting in a chronic sleep debt and a cumulative increase in homeostatic sleep pressure.^45^ Our overall study sample of participants with schizophrenia self-reported a median of 8 hours of sleep per night, which lies within the normal range,^32^ but over 3 quarters reported experiencing insomnia. Although, without adjustment, the prevalence of major cardiovascular events among participants with insomnia, very short and short sleep duration was raised, subsequent QRISK3 adjustment attenuated these findings. Previous studies in the general population have found daytime sleepiness to be independently associated with increased risk for cardiovascular mortality^46^ and stroke.^47^ It is unclear as to what the underlying mechanism causes this association, but one potential explanation is that daytime sleepiness may be associated with overall poorer health.^46^ In our study, a higher proportion of participants with schizophrenia and major cardiovascular events had been diagnosed with type 2 diabetes and had higher blood pressure compared to participants without major cardiovascular events. These variables, however, are included in the QRISK3 score, which we adjusted for.

Daytime sleepiness is an easily recognized and manageable condition. Light has an important function in regulating circadian rhythms.^48^ Interventions involving exposure to light scheduled during the day, such as a walk in the morning, impacts circadian rhythms and provides physical activity.^49^ Many patients with schizophrenia lack structure to their day, which can lead to a lack of motivation to get out of bed and increase daytime naps, thus reducing sleep pressure.^49^ Additionally, pro-dopaminergic medications such as modafinil have been trialed in the treatment of negative symptoms of schizophrenia, including daytime sleepiness, but studies conducted to date have not yielded a consensus on potential for benefit due to methodological issues.^50,51^

A second finding of our study is that the prevalence of major cardiovascular events was higher among people with schizophrenia with low muscular strength. Prevalence ratios remained significantly raised after adjustment for QRISK3 score. Evidence from the general population indicates an association between muscular weakness and elevated risks for multiple CVD outcomes.^25^ In our sample of participants with schizophrenia, mean muscular strength was substantially lower than UK sex- and age-specific normative data.^44^ Therefore, our findings suggest that it is especially important to measure and address muscular weakness in clinical practice for patients with schizophrenia. Muscular strength has been used as a proxy to determine general health and fitness.^25,52^ However, compared with measures of cardiorespiratory fitness and physical activity, muscular strength is easily measured objectively and cheaply^53^ and it provides clinical utility in community settings where blood sampling is not always feasible.^54^ Interventions that have combined aerobic and resistance training have demonstrated positive results in increasing muscular strength for people with schizophrenia.^55^ Another study showed that resistance training provided sustained improvement in muscular strength and reduced systolic, diastolic and mean blood pressure among hypertensive elderly women.^56^

We found no difference in prevalence of major cardiovascular events among people with schizophrenia with or without sedentary behavior. This finding contradicts existing evidence for the general population, which indicates an association between sedentary behavior and the risk for CVD onset and subsequent related mortality.^24^

A major strength of this study is that we used data from the UK Biobank, which is a rich data source that contains well-measured information for the candidate risk factors of interest examined here, which is unavailable in routinely collected data such as primary care databases. Some limitations should, however, also be considered in the interpretation of our findings. As the study design was cross-sectional, causal inferences cannot be made because the temporal nature of the observed relationship cannot be elucidated from such a design.^57^ This prevents an evaluation of whether daytime sleepiness and muscular weakness are predictive of adverse cardiovascular outcomes or have arisen as a consequence of such outcomes. In the general population, daytime sleepiness may be present in as many as a third of patients post-MI^58^ and may be independently predictive of poor prognosis.^59^ Similarly, in the general population, muscular weakness is more common among patients with CVD compared with control patients who were unaffected by CVD,^60^ and grip strength may predict cardiac death among patients with cardiac disorders.^61^ This suggests reducing daytime sleepiness and improving muscular strength is still of importance even if causality cannot be demonstrated or assumed. However, intervening early in the course of schizophrenia, such as during the first year of treatment, has been shown as a critical period in preventing premature cardiovascular mortality in this group.^62,63^

The UK Biobank recruited 503 325 persons aged 40–69 years, but the participation rate was only 5.5%, meaning a selection bias in favor of people willing to volunteer. Therefore, participants in our sample may be more highly functioning than the population of all persons diagnosed with schizophrenia. Thus, our study may have underestimated prevalence of major cardiovascular events, self-reported sleep disturbance, and sedentary behavior. In addition, face-to-face assessments are not used when making diagnoses of mental health disorders in the UK Biobank. Instead, the diagnoses of schizophrenia and related disorders in this study were identified via linkage of the UK Biobank participants’ hospital records (Hospital Episode Statistics [HES]). There is, however, evidence that use of linked data for diagnoses of schizophrenia spectrum disorders is valid and reliable.^64^

## Conclusions

Prevalence of major cardiovascular events appears to be higher among people with schizophrenia who have muscular weakness and sleep disturbance, evidenced by daytime sleepiness, independent of other known risk factors. Further consideration of these factors is warranted in clinical risk assessments for CVD in this vulnerable group. Future research should therefore establish how these can be properly accounted for in bespoke risk assessment tools for use in SMI populations. Furthermore, given the independence of the risk factors identified here, even beyond established factors, further efforts are required to address these often-neglected aspects of SMI in mental healthcare settings.

## Funding

This study was funded by the Medical Research Council and University of Manchester as part of AB’s MRC Doctoral Training Scholarship. ARY is supported by a National Health and Medical Research Council Principal Research Fellowship and National Institute of Health Research Senior Research Fellowship.
